# ROSPaCe: Intrusion Detection Dataset for a ROS2-Based Cyber-Physical System and IoT Networks

**DOI:** 10.1038/s41597-024-03311-2

**Published:** 2024-05-10

**Authors:** Tommaso Puccetti, Simone Nardi, Cosimo Cinquilli, Tommaso Zoppi, Andrea Ceccarelli

**Affiliations:** 1 Department of Mathematics and Informatics, University of Florence, Viale Morgagni 67/a, 50134 Firenze, FI Italy; 2 Mermec Engineering, Via Livornese 1019, 56122 Pisa, Italy; 3 Department of Engineering and Information Science, University of Trento, Via Sommarive 9, 38124 Trento, Italy

**Keywords:** Scientific data, Computer science

## Abstract

Most of the intrusion detection datasets to research machine learning-based intrusion detection systems (IDSs) are devoted to cyber-only systems, and they typically collect data from one architectural layer. Often the attacks are generated in dedicated attack sessions, without reproducing the realistic alternation and overlap of normal and attack actions. We present a dataset for intrusion detection by performing penetration testing on an embedded cyber-physical system built over Robot Operating System 2 (ROS2). Features are monitored from three architectural layers: the Linux operating system, the network, and the ROS2 services. The dataset is structured as a time series and describes the expected behavior of the system and its response to ROS2-specific attacks: it repeatedly alternates periods of attack-free operation with periods when a specific attack is being performed. This allows measuring the time to detect an attacker and the number of malicious activities performed before detection. Also, it allows training an intrusion detector to minimize both, by taking advantage of the numerous alternating periods of normal and attack operations.

## Background & Summary

*Intrusion Detection Systems*^1^ (IDSs) are of critical importance to secure systems against attacks. They consist of two major components: a monitor and a detector. The first collects data from a running system, while the second analyses these data to discern between legitimate (normal) or malicious behaviors. While most commercial intrusion detectors rely on static rules, anomaly detection^2^ approaches based on machine learning constitute an attractive and widely researched alternative. They can develop complex decision criteria to classify each data point either as *normal* or *attack*, with also the ability to identify zero-days^3^, i.e., attacks never observed before and not yet described by static rules. An anomaly-based intrusion detector finds patterns in data that do not conform to the expected behavior of a system (or a network): these patterns, called anomalies^2^, are suspected attacks. Anomaly-based intrusion detectors are built under the assumption that ongoing attacks generate observable anomalies in the trend of the monitored performance indicators, or features, of the system^4^, network^5^, or both^6^.

To successfully train and test an anomaly-based intrusion detector, it is important to use realistic datasets specific to the operational domain and with analogous features and distribution concerning real-world data. The intrusion detection datasets available at the state-of-the-art mostly focus on cyber-only systems, while examples with cyber-physical systems are exceedingly scarce. In addition, these datasets are usually focused on monitoring the network traffic, without providing useful data to observe the manifestation of anomalies in other architectural layers. Also, the network traffic in the absence of attacks is often generated by modeling user profiles and simulating the user’s behavior: the normal and the attack behavior are unrelated because they are collected separately, ultimately preventing the datasets from capturing the dynamics of transiting from an attack-free state to an under-attack state. These pitfalls evidence the lack of intrusion detection datasets that accurately describe the behavior of a cyber-physical system in a real execution environment.

In this paper, we introduce the ROSPaCe^7^ (Robot Operating System 2 for Smart Passenger Center) dataset for intrusion detection, which is obtained by monitoring the SPaCe (Smart Passenger Center) embedded cyber-physical system. ROSPaCe^7^ aims to overcome the limitations stated above. Aside from the focus on the SpaCe system, the ROSPaCe^7^ dataset offers significant utility for the research in intrusion detection in connected domains, for example when studying attacks against IoT networks and cyber-physical systems.

The SPaCe system orchestrates onboard and ground components of a tramline carriage to optimize public mobility. It collects data from onboard cameras and processes them to improve the quality of the service, for example facilitating fleet optimization, supporting passengers, and commanding maintenance intervention. The system is built using ROS2, a popular protocol for robotic and distributed systems^8–12^. The operational capabilities of the SPaCe system impose the application of security solutions. While IDSs are particularly suitable for the context, they cannot be viably applied without relevant training data.

We perform attacks through the execution of discovery and DoS attacks, for a total of 6 attacks, with 3 of them specific to ROS2. We collect data from the network interfaces, the operating system, and ROS2, and we merge the observations in a unique dataset using the timestamp. We label each data point indicating if it is recorded during the normal (attack-free) operation, or while the system is under attack. The dataset is organized as a time series in which we alternate sequences of normal (attack-free) operations, and sequences when attacks are carried out in addition to the normal operations. The goal of this strategy is to reproduce multiple scenarios of an attacker trying to penetrate the system.

The final version of ROSPaCe^7^ includes above 30 million data points with 482 features, collected over 4 days of operation and for a total of approximately 38 GBs. We also present a simplified version of the dataset, which differs in the number of features and consequently has a reduced dimension of 14.5 GBs. We evaluate the performances of the IDSs using the accuracy, ROC curve, and precision-recall curve. Also, we show how ROSPaCe^7^ can be exploited to measure the attack latency at the cost of a maximum acceptable false alarm rate. In fact, in practice, a system developer is interested in minimizing the attack latency (identify an attacker as soon as possible), while maintaining false alarms reasonably low to avoid too many unnecessary attack responses. This is not achievable with most of the state-of-the-art datasets, because the dynamics of transiting from an attack-free state to an under-attack state are insufficiently captured.

In summary, the relevant features of ROSPaCe^7^ are: (i) it focuses on a cyber-physical system and IoT networks, being the first intrusion detection dataset targeting ROS2; (ii) it monitors multiple architectural layers, not being limited to logging network traffic; (iii) it is recorded from a real system under execution, so that there is no simulated traffic or simulated user’s profile; (iv) it is composed of multiple distinguishable scenarios, modeling an attacker attempting to penetrate a clean system while it is operating. The validation results suggest ROSPaCe^7^ may become a valuable resource for training and evaluating IDSs for ROS2-based systems and, broadly, a useful dataset to develop and evaluate IDS solutions to embedded cyber-physical systems and IoT networks.

Noteworthy, the attacks successfully targeted the system and degraded the system’s performance: we remark that the SPaCe system has been successively modified to implement defenses, and the attacks are no longer effective against the deployed system.

## Related Works: Intrusion Detection Datasets

In this section, we report on the most used public datasets for intrusion detection with the help of Table 1, and we elaborate on the differences from ROSPaCe^7^.

**Table 1:** Summary of the most famous dataset for intrusion detection, and comparison with ROSPaCe7.

dataset name	year	environment	normal traffic	features type	number of features	millions of data points	application-specific attacks	attacker dynamics	accuracy
KDDCup99^13^	1999	real	conventional	network	41	4.9	no	yes	0.85
NSL-KDD^13^	2009	real	conventional	network	41	4.9	no	yes	0.84
ADFA-LD^18^	2014	real	conventional	Syscall	3 792	2.7	yes	no	0.90
ADFA-WD^18^	2014	real	conventional	syscall	4 801	205.6	yes	no	0.91
ISCX^14^	2012	real	simulated	network	20	0.6	no	yes	0.87
CICIDS17^15^	2017	real	simulated	network	80	0.2	no	yes	0.89
CICIDS18^15^	2018	virtualized	simulated	network	80	162	no	yes	0.98
InSDN^20^	2020	virtualized	simulated	network	56	0.2	yes	no	0.95
IoT-IDS^16^	2019	real	simulated	IoT	8	0.3	no	yes	0.96
ROSPaCe^7^	2023	real	conventional	network, OS, ROS2	60–482	30.2	yes	yes	?

The table reports (i) the name of the dataset, (ii) the release year, (iii) the target environment, distinguishing from real systems or virtual networked systems, (iv) if the normal (attack-free) operativity corresponds to the nominal activities of the deployed system (*conventional*) or is stimulated with synthetic user profiles (*simulated*), (v) the type of features collected, identifying the architectural layers that are monitored, (vi) the number of features monitored, (vii) the number of data points, (viii) the availability of attacks specific to the system, software, and application, in contrast to attacks to common network services, (ix) if the dataset provides alternating patterns of normal behavior and periods under attack, to model the advent of an attacker in an attack-free system, or if the normal and attack behaviors are collected separately, and (x) the accuracy of state-of-the-art supervised algorithms, obtained from past research works (accuracy of KDD Cup99^13^, NSL-KDD^13^, ISCX^14^, CICIDS17^15^, and IOT-IDS^16^ is from Thakkar *et al*.^17^, the accuracy of CICIDS18^15^ is from Leevy *et al*.^1^, the accuracy of ADFA-LD^18^ is from Khandelwal *et al*.^19^, the accuracy of InSDN^20^ is from Negera *et al*.^21^, and the accuracy of ADFA-WD^18^ is from Khraisat *et al*.^22^).

Different from all the reviewed datasets, ROSPaCe^7^ provides features from multiple architectural layers (Linux, ROS2, network), and it is the first intrusion detection dataset that monitors ROS2 features. This focus on ROS2 stands also for the attack selection because we provide 3 different attacks specific to the exploitation of known ROS2 vulnerabilities. Furthermore, the dataset collects all traffic during the system’s nominal execution, rather than simulating the normal usage of the system with a synthetic user profile.

Lastly, the ROSPaCe^7^ dataset describes the dynamic of an attacker trying to exploit the system, by providing observation in a time-series form where periods of normal execution and a single attack are alternated. By marking the start of attacker operations, we can measure the shortest time an attacker is discovered once it starts its attack sequence. This allows assessing IDS detection capabilities not only based on accuracy but also on a broader understanding of how fast the detector can recognize an attack. For example, notorious datasets like CICIDS17 are composed of long periods of normal operativity, followed by long periods where different attacks are injected. In this way, the scenario of an attacker entering the system and performing actions on an “attacker-free” system is insufficiently captured.

## SPaCe system and ROS2

The SPaCe system is designed to orchestrate and optimize public mobility, improving the user experience and preventing security violations. It performs surveillance of tram carriages using on-board cameras, to determine the operative conditions of the tram carriage in real-time. SPaCe analyses the video stream from cameras to establish the occupation of the vehicles and notify users and administrators. It also provides detection capabilities to request intervention in case of damage to the vehicle and its equipment.

The SPaCe system is structured into 3 basic components. A remote server orchestrates the fleet and dispatches information to the users and the administrators. The onboard architecture of each tram carriage collects, processes, and anonymizes the data from the onboard cameras so that it can be sent to the remote server for fleet optimization and coordination. Finally, user devices show the information to the end users, with different interfaces based on the user privileges.

**Fig. 1:**
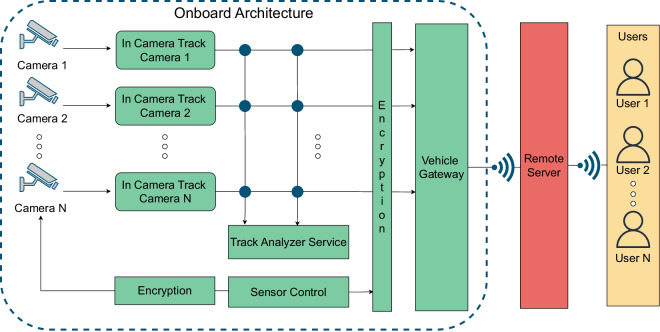
The SPaCe architecture, with a focus on the on-board architecture. It is a cyber-physical embedded system where video cameras monitor the tram carriage, and the information is locally processed through machine learning algorithms. Elaborated and anonymized information is then transmitted to the remote server.

In this paper, we focus on the tram carriage and its onboard architecture. We describe the architecture of the onboard system with the aid of Fig. 1. A camera provides a video stream to the *In Camera Track* module. This module, in turn, makes the first processing of the video stream. The In Camera Track identifies the relevant elements within the scene and reconstructs their space-time consistency. This module is composed of a set of microservices: (i) the *image manager* implements the data flow management: it interacts with the camera sensors by extrapolating the raw data and normalizing the data structure; (ii) the *detector* allows the extraction of features relevant to the analysis of the status of vehicles and stations, e.g., the presence of people, object classification, and position assessment; (iii) the *tracker* tracks a target object or person in successive frames.

The In Camera Track sends the elaborated information to the *Track Analyzer Service*. This module extrapolates the information useful to the SPaCe system, for example, if there are lost objects, broken items, dangerous situations, as well as status information on the capacity of the vehicle. More precisely, the Track Analyzer Service implements a set of microservices that provide insight into the traces detected by the trackers. Its main role is to reconstruct the information necessary for a posteriori analysis of the track. This module includes the following microservices: (i) the *face and clothes attributes* extract the biometric information needed for facial recognition, and profiles users based on their clothes; (ii) the *human keypoints* analyses the passenger movements a posteriori differentiating between legit or forbidden behavior; (iii) the *object attributes* extract the attributes from the objects, to allow the subsequent recognition in a completely anonymous way. The information is then transmitted to the remote server. Lastly, the *Sensor Control* module encrypts and stores the raw data from the camera, which is available upon request.

The prototype of the SPaCe onboard architecture we are using is installed and operated at Mermec premises. The implementation relies on ROS2^10–12^, an open-source software platform for developing robotic applications. ROS2 is largely applied in embedded cyber-physical systems^8,9^, thanks to its ability to deploy and coordinate distributed microservices. ROS2 applications consist of independent computing processes called nodes that communicate in a publish/subscribe fashion, passing messages via a topic: a node publishes a message to a topic, and topic subscribers can utilize the message^11^. ROS2 also provides a communication paradigm alternative to publish/subscribe: a node can expose a service that can be exploited directly by another node, in a remote procedure call fashion. Unlike ROS, which relies on a centralized master process to handle communications, ROS2 exploits the standard Data Distribution Service^12^ (DDS) which is completely distributed. The DDS introduces procedures to optimize the Quality-of-Service (QoS), especially to optimize the application for the available bandwidth and latency and to increase guarantees on message delivery. In SPaCe, the ROS2 *foxy* version is available as a middleware service on top of the Linux Ubuntu 20.04 operating system.

## Methods

We present the experimental setting and the steps to compose the dataset. The discussion is divided into three parts. First, we define our system and its setting, including the monitoring system, the data collection method, and the attacks towards the on-board architecture. Second, we present the data processing and labeling to create a unique dataset from traces collected by different monitors. Third, we describe the resulting datasets, and we reduce the set of features to provide a lighter version of the dataset.

### Part 1: Experimental Set Up

**Fig. 2:**
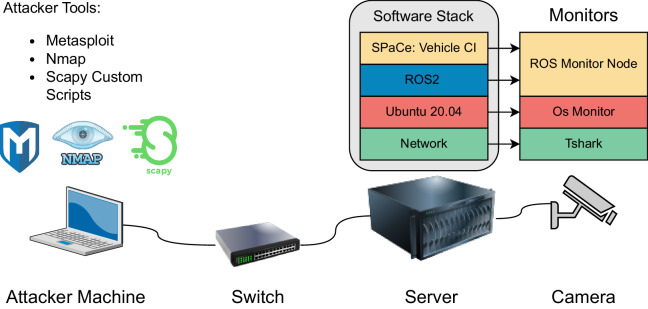
The setting of our experimental campaign. The Ubuntu 20.04 Server runs ROS2, and it is connected to one camera sensor via a LAN cable. The attacker operates from an Ubuntu 20.04 laptop connected via a switch. The attacker tools are Metasploit, Nmap, and custom scripts implemented using the Scapy library. The software probes monitor data from three architectural layers: the network, the operating system, and the ROS2 middleware and microservices.

We describe the experimental setup with the aid of Fig. 2. Our experimental set-up is composed of (i) the onboard architecture, which is a server running Linux Ubuntu 20.04 and ROS2 foxy, (ii) a camera connected via LAN cable to the server, and, (iii) the attacker machine, which is an Ubuntu 20.04 laptop connected to the onboard architecture through a switch.

Monitors are installed on the server, to observe features from the network layer, the operating system layer, and ROS2. The data collection system requires the contribution of the attacker machine, and the server as will be discussed later.

#### Attacks

We represent a scenario where an attacker can launch attacks towards the server: we assume the attacker is aware of the IP address of the Ubuntu server. This could happen, for example, if the attacker can exploit the communication channels between SPaCe components. To run and implement the attacks, we rely on different tools installed on the attacker’s machine, namely Nmap, Metasploit, and custom scripts we developed. The network mapper Nmap^23^ is a free and open-source utility for network discovery and security auditing. Nmap uses raw IP packets to determine the available network hosts, the services (application name and version) and operating systems being executed on the target hosts, the type of packet filters and firewalls in use, and many other characteristics. We run Nmap version 7.93. Metasploit^24^ relies on a large central repository of known exploits. The user can select an exploit from the central repository, and the payload to run after the breach. We rely on the PyMetasploit^25^ library to integrate and run Metasploit in a Python program. Lastly, we select scripts from the Robot Hacking Manual^26^ that are specific to attack ROS2, and we customize them to fit our experimental setting. These scripts rely on Scapy^27^, a powerful interactive packet manipulation library in Python. Scapy can forge or decode packets of a wide number of protocols, send them on the wire, capture them, and match requests and replies.

We select attacks using as reference an existing ROS2 threat model^28^. Consequently, we focus on attacks that exploit the external network, especially the network and the ROS2 DDS as they are highlighted among the main entry points. The attacks are from two categories, usually termed *discovery*, and *denial of service* (DoS). A discovery attack consists of collecting information about the target system, rather than exfiltrating sensible data or directly damaging the system. While they do not directly compromise the system, these attacks provide information that can be used by attackers to breach system defenses. Instead, DoS is aimed at reducing or interrupting the availability of a service exposed by the target system. An attacker can implement a DoS in multiple ways, targeting different parts of the system stack, still making the system’s services unavailable.

Overall, we select 2 discovery attacks and 4 DoS attacks. Three attacks are generic and provided by state-of-the-art tools, and 3 are specific to the ROS2 DDS.

#### Discovery attacks

*Nmap Discovery* is mainly used to extrapolate the version of the operating system of the target machine. It relies on an internal database of fingerprints collected from a large variety of operating systems. It builds a fingerprint of the unknown system by analyzing the responses to crafted TCP and UDP requests. Then it searches for a complete or a partial matching of the fingerprint in the database. We use the Nmap OS Detection script^23^ to run the attack with the command *nmap -O –osscan-guess*.

*ROS2 Reconnaissance* is a discovery attack specific to ROS2 that extrapolates information about the running nodes and their services. When the usage of a simple ROS2 query is not possible, it tries to exploit the vulnerabilities of the DDS by sending a specifically forged package. A vulnerable DDS will respond with a service message that lists the running nodes on the ROS2 architecture. We exercise the attack relying on the code from the Robot Hacking Manual^26^.

#### DoS attacks

*Flooding Meta* is the Metasploit implementation of the common network-level SynFlood attack. It consists of sending many packets with the SYN bit = 1 to request a new connection to the target machine. The target system replies with SYN-ACK packets, allocates resources to handle the connection, and waits for an ACK packet that confirms the connection. The attacker ends the interaction in this state causing the system to waste computational and memory resources. The script that implements the attack is *auxiliary/dos/tcp/synflood*^24^ available in Metasploit.

*Flooding Nmap* is the Nmap implementation of a network-level DoS attack, that we exercise relying on the *ipv6-a-flood.nse*^23^ script.

*ROS2 Node Crashing* exploits known vulnerabilities of some versions of the DDS which cause incorrect handling of ROS2 messages that do not respect the declared length. Like in buffer overflow attacks, the attacker can cause the execution of a piece of code hidden in the packet or can lead to a crash of the target ROS2 node. This attack is implemented with a custom Python script provided by the Robot Hacking Manual^26^ and written using Scapy.

*ROS2 Reflection* exploits a vulnerability of the communication protocol used by the DDS. It consists of modifying the multicast address of a ROS2 node by sending a purposely crafted message. If successful, the attacker can redirect the node to listen to messages from a malicious server. The server produces continuous traffic to saturate the resources. The attack is implemented using Python and Scapy and the implementation is provided by the Robot Hacking Manual (RHM)^26^.

#### Monitors

We describe the software probes that we set up to monitor the system execution. The probes are installed on the server as shown in Fig. 2.

*Tshark*^29^ is a well-known tool for monitoring network activity. It targets the network layer, and it is configured to collect all the packets transiting the network interface. The collected packets are logged in*.pcap* files for later processing. The list of the 451 monitored features is not described here for brevity, but it is available as a.*xlsx* file in the dataset repository (*feature_list.xlsx*).

The *OS Monitor* is a monitoring software we built to target the OS layer of a Linux machine. It collects all the information provided by the */proc* virtual filesystem^30^, such as memory usage, kernel configuration, installed devices, the status of the CPU cores, and information specific to each process running on the OS. The OS monitor operates with a period of 200 milliseconds. In Table 2, we list the 25 features that we monitor from the operating system, along with their description.

**Table 2:** Monitored features of the virtual filesystem /proc/.

Indicator Name	Description
MemFree	The amount of RAM, in kilobytes, left unused by the system.
Buffers	The amount of RAM, in kilobytes, used for file buffers.
Cached	The amount of RAM, in kilobytes, used as cache memory.
Active	The total amount of buffer or page cache memory, in kilobytes, that is in active use. This is memory that has been recently used and is usually not reclaimed for other purposes.
Inactive	The total amount of buffer or page cache memory, in kilobytes, that are free and available.
SwapFree	The total amount of swap-free memory, in kilobytes.
Pgpgin	Number of pageins (since the last boot).
Pgpgout	Number of pageouts (since the last boot).
pgalloc_dma	Number of page allocations per zone (since the last boot).
Pgfree	Number of pagesfrees (since the last boot).
Pgactivate	Number of page activations (since the last boot).
Pgdeactivate	Number of page deactivations (since the last boot).
Pgfault	Number of minor page faults (since the last boot).
Pgmajfault	Number of major page faults (since the last boot).
Disk_Read	read operations executed on disk since the last observation.
Disk_Write	write operations executed on disk since the last observation.
Net_Received	Bytes of data received from the network since the last observation.
Net_Sent	Bytes of data sent through the network since last observation.
Tcp_Listen	Number of listening TCP sockets.
Tcp_Established	Number of active TCP sockets.
Tcp_Syn	Number of synchronized TCP sockets.
Tcp_TimeWait	Number of waiting TCP sockets.
Tcp_Close	Number of closed TCP sockets.
nr_active_file	Number of active files
nr_inactive_file	Number of inactive files

The *ROS Monitor* is a custom node that we developed to monitor the ROS2 layer. The node subscribes to each topic published by the active nodes. The ROS Monitor scans periodically for new nodes to monitor changes in the system that may occur over time. The lists of monitored nodes include the /*rosout* node which is the default logger of the ROS2 architecture and collects logging data from the active nodes. Implementing the ROS Monitor node avoids directly using the /*rosout* logging operation, which would create a *bag file* to save every ROS2 message. Producing a bag file is invasive and burdensome, meaning that it is too intrusive for monitoring purposes. This limits the number of features we get from the ROS Monitor. However, we believe that these metrics are semantically relevant to describe the behavior of the ROS microservices. In particular, they quantify information about the communication patterns and the interactions observed in the ROS publisher/subscriber paradigm. The ROS monitor operates with a period of 200 milliseconds. In Table 3 we report the 5 features obtained from the monitor along with their descriptions.

**Table 3:** Monitored features of the ROS Monitor Node.

ROS2 Feature Name	Description
src_topic	Indicates the source node of a specific message.
subscribers_count	Number of subscribers to a topic.
publisher_count	Number of publishers to a topic.
msg_type	Type of a ROS2 message.
msg_data	The header of a ROS2 message.

It collects information from each topic including /rosout which is the default logging system in ROS2.

#### Experimental campaign

**Fig. 3:**
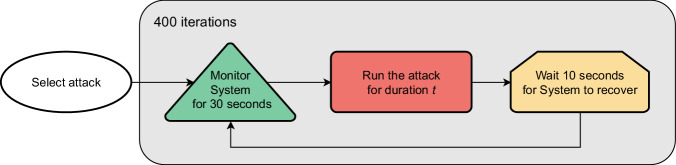
The script that automates the execution of the experimental campaign.

Once the onboard server and the monitors are up and running, we execute a custom Python script on the attacker machine, to coordinate and automate the experimental campaign. The script, schematized in Fig. 3, iteratively performs the following actions, for the number of attacks:

1. Select an attack *x* that was never selected before.
2. Wait 30 seconds, without performing any action. In this period, the target system is behaving normally, in an attack-free scenario.
3. Inject the attack *x*, for a maximum temporal duration *t*.
4. Stop injecting *x* and keep the system in a hiatus state for 10 seconds. This period is set to allow the system to restore a clean state. We expect that after this period, the effect of the attack will no longer manifest on the monitored features. In case services are crashed or unable to recover, the system is rebooted, and the experimental campaign restarts from step 2.
5. Repeat steps 2, 3, and 4 for 400 iterations. This means that 400 sequences of normal operations and successive injections of attack *x* will be logged.
6. Wait 90 seconds to restore a clean system state, then restart from 1.

We set the temporal duration *t* = 60 seconds for Flooding Meta and Flooding Nmap attacks, after which the attack is interrupted. The duration of the other attacks depends on many factors and can change at each run, with a timeout of *t* = 60 seconds. The waiting time of steps 4 and 6 is introduced to allow system recovery after an attack, such that the value of the monitored features is no longer altered by the attack.

All the steps above are commanded by the attacker machine, and locally timestamped and logged.

### Part 2: Data Processing and labeling

At the end of the experimental campaign, we obtain three logs from our three monitors for a total of 27.3 GB. The ROS monitor and the OS monitor log in CSV format, while Tshark records.pcap files. We export the.pcap files collected from Tshark in a.*json* format, generating multiple files of about 10 GB each, and then we process the.json files with the Pandas^31^ library to obtain CSV files.

To merge the CSV files from the 3 different monitors, we use the timestamp as a key, and as a reference the CSV file obtained from Tshark. The reason is that Tshark, differently from the other monitors which logs with a period of 200 milliseconds, is not configurable with a sampling interval and collects any packet. Consequently, the Tshark CSV file contains observations at much shorter intervals compared to the other monitors. We concatenate each row from the Tshark CSV file with rows of the OS monitor and ROS monitor, if their timestamps differ by less than 100 milliseconds (ms). Conversely, if the difference between the timestamps is greater than 100 ms, the rows are not concatenated. This algorithm works given the logging period of 200 ms for both the ROS Monitor Node and the OS monitor, with some exceptions related to the ROS Monitor Node due to successful attacks that crash the entire ROS2: in such cases, there are no data from the ROS Monitor Node. Due to computational constraints and the file size, we perform this operation by splitting the CSV file into batches.

To label each data point (each row) of the resulting CSV file, we rely on the log of the attacker machine. The attacker machine locally logs a timestamp at each step of the experimental campaign. We use these timestamps to identify (i) the start of the 30 seconds of normal operation; (ii) the start of the successive attack period; and (iii) the termination of the attack period. This way, we can identify data points produced in the absence of attacks, that we label as *normal*, and data points produced when a specific attack is exercised, that we label with the name of the attack. Data collected during the restoration periods are discarded.

**Fig. 4:**
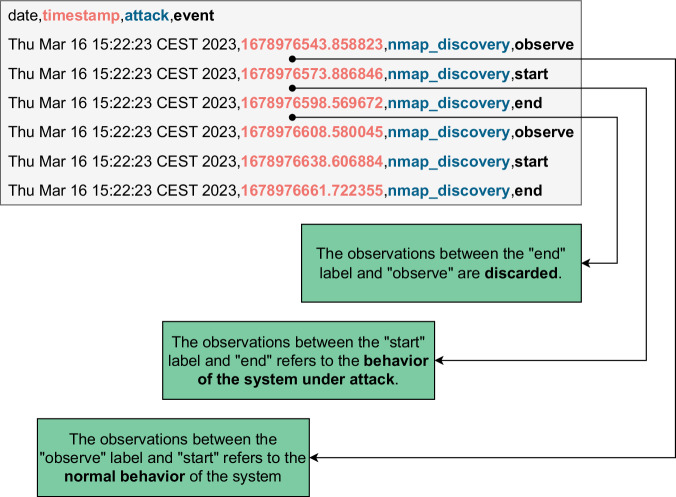
The observations between “start” and “end” are labeled with the attack name, while the observations between “observe” and “start” are labeled as normal. We discard the observations between the “observe” and “start” labels because they are collected while the system is recovering to a clean state.

Figure 4 schematizes the labeling procedure. We label the observations between the “observe” and the “start” timestamps as normal, and we label the observations between the “start” and the “end” timestamps with the name of the attack. We discard the observations between the “observe” and “start” labels because they are collected after an attack, while we are allowing the system to recover. With this procedure, we identify observations collected during the temporal execution of the attacks, with the only approximation of time synchronization between the two nodes, which are synchronized to a global time server using the Network Time Protocol^32^.

We remark that we do not indicate when the effect of a given attack manifests on the monitored features, but we indicate when the attack session starts. In other words, while we can identify when the first attack packet is launched, we cannot tell when (or if) the attack will affect the value of the monitored features and consequently may be visible to anomaly detection.

Last, we observe that the features of the data points logged by Tshark may differ, depending on the traffic. This means that the number of features logged may vary from one data point to another, with some features that can be observed only in very few messages. To facilitate its usage by machine learning algorithms and avoid empty fields, we select only the features that are in common between all files, by concatenating the CSV file batches using an inner join operation on the columns. Further, we delete: (i) features whose values are linear indexes, that simply identify the order of data points; (ii) duplicate features; (iii) features with only 1 value. At the end of this process, the number of features is 482, excluding the label. The recorded.pcap files from Tshark are available on the repository^7^.

## Data Records

### ROSPaCe dataset

**Fig. 5:**
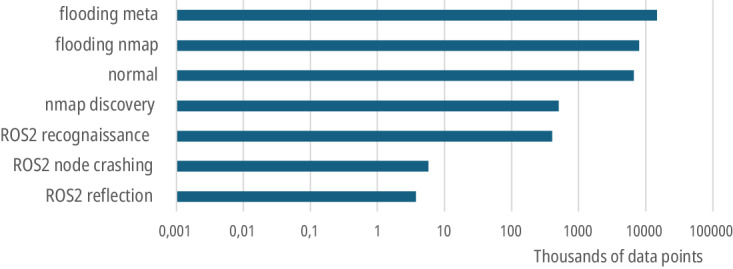
Number of attack data points and normal data points. The x-axis is in logarithmic scale.

The ROSPaCe^7^ dataset has been deposited and is freely available. The final shape of the ROSPaCe^7^ dataset is 30 247 050 data points and 481 columns excluding the label. The features are 25 from the OS monitor, 5 from the ROS Monitor Node, and 452 from Tshark. The dataset is encoded in the *ROSPaCe_complete.csv* file for a total of 38 GB. The dataset contains about 23 million attack data points and above 6.5 million normal data points (78% attacks, 22% normal). We specify in Fig. 5 the number of observations collected. Reasonably, DoS attacks have the highest number of attack data points. Instead, the low number of data points of *ROS2 Reflection* and *ROS2 Node Crashing* is because the attacks lead to the failure of one or more ROS2 nodes, in some cases including the monitors. Note that these cases required a complete restart of the system and an interruption of the experimental campaign. This is also confirmed in Table 4, where the left part describes the temporal duration of the attack sequence, for each attack type, in terms of average, minimum, and maximum duration, while the right part describes the average, minimum, and maximum number of data points in the attack sequence.

**Table 4:** Left: average, minimum, and maximum temporal duration of attack sequences, in seconds.

attack	average duration	minimum duration	maximum duration	average length	minimum length	maximum length
flooding meta	60.97	59	160	43 303	812	171 799
nmap discovery	22	20.45	24.66	1 377	1 180	8 248
flooding nmap	29.86	29.80	29.95	398 655	467 044	40 534
ROS2 node crashing	1.05	0	40.23	51	2	150
ROS2 reconnaissance	13.14	9.36	60.16	1 907	1 295	4 873
ROS2 reflection	3.69	0	40.20	24	1	99

Right: average, minimum, and maximum number of data points in an attack sequence.

### ROSPaCe reduced dataset

We provide a lightweight version of the ROSPaCe^7^ dataset by selecting the best-performing 60 features (aside from the timestamp and the label). This includes the 30 features from the OS monitor and the ROS monitor and the 30 best-performing features from Tshark. The reduced dataset is smaller than the ROSPaCe dataset only in terms of the number of columns, while the data points are the same.

We proceed by inspecting the dataset to identify and remove those that may reduce the model’s ability to generalize, potentially leading to a degradation in detection performance. For example, these are features that specify the source or destination at different layers of the protocol stack. Then, we reduced the Tshark features by selecting the 30 most important ones using Extremely Randomized Trees^33^ meta-estimator. This meta-estimator fits a fixed number of randomized decision trees on a subset of samples of a dataset and uses averaging to select the features that contribute the most to the classification, i.e., identifies the features that are mostly used to make classification decisions.

The ROSPaCe reduced dataset is split in 15 CSV files of 913 Mb each for a total of 14.5 GB (*ROSPaCe_reduced.tar.gz*^7^).

### ROSPaCe no-periodicity dataset

We generate a modified version of the dataset where sequence durations are randomly adjusted, and timestamps are not absolute values (clock readings) but are relative to each sequence. This methodology preserves the dynamics between normal behavior and attacks within sequences while preventing time series models like LSTM^34,35^ from exploiting timestamp periodicity as a label, to detect attacks. In particular, we operate as follows:

1. We identify each sequence in the dataset.
2. For each sequence, we cut the initial data points, to an amount between 1% and 35% of the normal data points in the sequence. The value is chosen randomly for each sequence.
3. For each sequence, we cut the final part of the sequence, i.e., the attack data points, to an amount between 1% and 70% of the attack data points in the sequence. Again, the value is chosen randomly for each sequence. The percentage is higher concerning normal data points, to reflect the overall balance between normal and attack data points in the dataset.
4. We offset the timestamp features of the sequence, to make the sequence start at 01-01-23 00:00:00. This way, the timestamps are relative to each sequence rather than to the complete dataset.

The no-periodicity dataset *ROSPaCe_complete_noperiodicity.csv* is given as an additional resource^7^. Further, we realize a reduced version of the no periodicity dataset, namely *ROSPaCe_reduced_noperiodicity.tar.gz*^7^ applying the same methodology reported for the ROSPaCe reduced dataset. The *ROSPaCe_reduced_noperiodicity.tar.gz* archive contains and compresses the *ROSPaCe_reduced_noperiodicity.csv* file, which is split into 10 files, each with a size of 1 GB.

## Technical Validation

In this section, we show that the dataset is well-formed and can be used for intrusion detection purposes. We use the reduced dataset (*ROSPaCe_reduced.tar.gz*^7^) as it is easier to manage. First, we apply the feature ranking technique to quantify the correlation between features and labels. Then, we apply visualization techniques to graphically understand if the distribution of normal data points and attacks is different and if there is a tangible partitioning between the classes. Last, we execute machine learning algorithms leveraging the two methodologies proposed in the Usage Notes.

### Feature ranking and data visualization

We compute the importance of features on the reduced dataset through mutual information^36,37^ and chi-square^38,39^ feature ranking strategies. Feature rankers quantify the correlation of each feature with the label: this allows identifying the features that are most informative to the label. Notably, these strategies are model-agnostic, ensuring unbiased assessment across machine learning models.

In Table 5 we show the results for the mutual information and the chi-square statistics. Interestingly, the two analyses produce separate lists of the top 5 features, some of which overlap but are ranked differently. The best features for the mutual info analysis are *pgpgout*, *src_topic*, and *layers.ipv6.ip.version* for respectively, the OS Monitor, the ROS Monitor Node, and Tshark. The best features selected by the chi2 statistic are *pgfree* (OS Monitor), *src_topic* (ROS Monitor Node), *layers.ip.ip.flags* (Tshark). Overall, the analysis demonstrates that the features included in the dataset have consistent power in differentiating normal behavior from attacks.

**Table 5:** Chi 2 score (chi 2) and mutual info score (mutual info) feature ranking.

	feature name	chi 2	feature name	mutual info
**OS**	Pgpgout	8.807 × 10^6^	Pgfree	1.15303
	Net_Received	7.155 × 10^6^	Pgfault	1.15301
	MemFree	6.427 × 10^6^	Pgpgout	1.15199
	Tcp_TimeWait	5.050 × 10^6^	Pgactivate	1.15098
	Pgfault	4.750 × 10^6^	Active	1.14925
**ROS**	src_topic	4.793 × 10^6^	src_topic	0.30932
	msg_type	2.066 × 10^6^	subscribers_count	0.25884
	publishers_count	1.547 × 10^6^	msg_type	0.24204
	subscribers_count	1.047 × 10^6^	publishers_count	0.24142
	msg_data	2.400 × 10^5^	msg_data	0.20714
**Tshark**	layers.ipv6.ip.version	2.150 × 10^7^	layers.ip.ip.flags	0.84398
	layers.icmpv6.icmpv6.type	2.077 × 10^7^	layers.tcp.tcp.stream	0.81332
	layers.ip.ip.flags_tree.ip.flags.df	1.369 × 10^7^	layers.tcp.tcp.window_size	0.79519
	layers.tcp.tcp.flags_tree.tcp.flags.syn	1.240 × 10^7^	layers.tcp.tcp.window_size_value	0.78927
	layers.tcp.tcp.flags_tree.tcp.flags.syn_tree	1.240 × 10^7^	layers.ip.ip.checksum	0.75566

We report for the Os Monitor (OS), ROS Monitor Node (ROS), Tshark the best 5 features ranked based on the chi 2 score (on the left) and the best 5 features based on mutual info score.

**Fig. 6:**
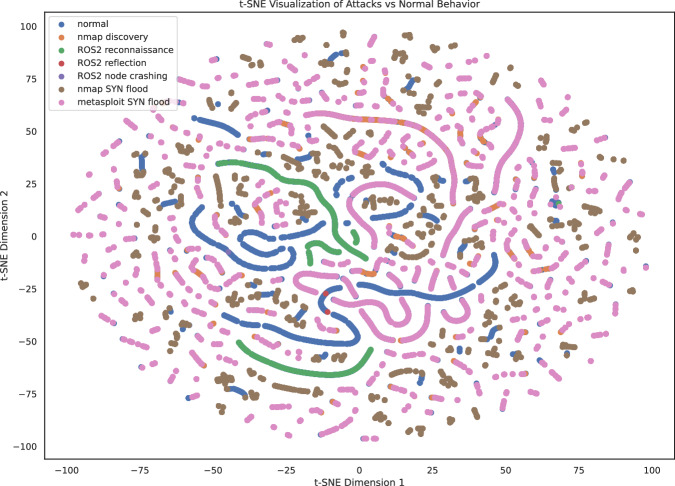
t-SNE visualization of normal or attack classes (painted in different colors) using *layers.ipv6.ip.version*, *pgpgout*, and *src_topic as features*.

Then, we apply t-SNE^40^ to visualize the high-dimensional data in two dimensions (Fig. 6). We launch t-SNE on the dataset by targeting the multi-label and using the chi 2 best-ranked feature from each of the 3 monitors: layers.ipv6.ip.version (Tshark), pgpgout (OS), and src_topic (ROS). To overcome computational limitations, we use 1% of data points, downsampled maintaining a uniform distribution. The t-SNE visualization shows some distinction between the normal data points (normal label in blue) and the attack data points, with some areas that are occupied exclusively by normal data points. However, many normal data points are covered by attack data points in the figure.

**Fig. 7:**
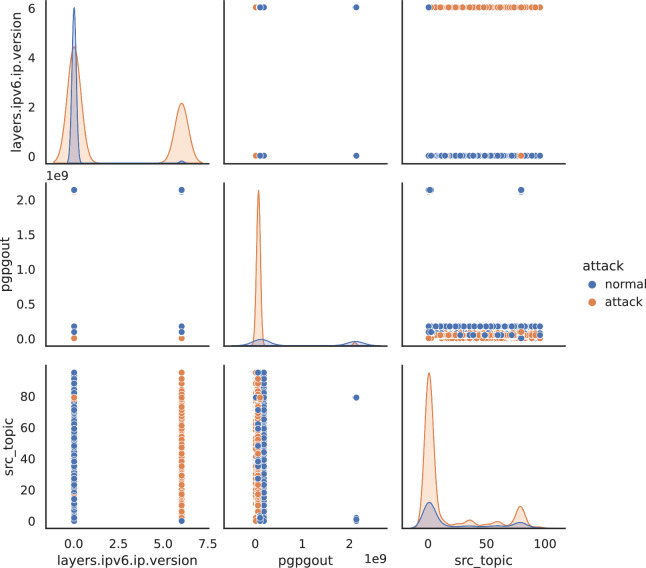
Seaborn scatterplot for the layers.ipv6.ip.version, pgpgout, and src_topic features. The diagonal plots show the distribution of each variable individually. The plots above the diagonal show the relationship between different pairs of variables.

Last, we plot a pairwise feature scatterplot matrix using Seaborn^41^. This visualization is useful for exploring relationships between pairs of features. We analyze the same features observed using t-SNE. In Fig. 7, we report the grid of scatterplots with 3 rows and 3 columns. The diagonal plots show the distribution of each variable individually. The plots above the diagonal show the relationship between different pairs of variables. For example, the plot at row 1, and column 2 shows how the *layers.ipv6.ip.version* feature relates to the *pgpgout* feature. The plots below the diagonal show the same relationships as above but are reversed (how *pgpgout* relates to the *layers.ipv6.ip.version*).

The scatterplot confirms the t-SNE analysis evidencing some overlapping between the normal data points distribution and the attacks distribution, but there is evidence of some distinction especially when the *layers.ipv6.ip.version* and the *src_topic* are considered jointly (upper right corner and, inversely, lower left corner). Figure 6 and Fig. 7 convey a similar message: attacks are often difficult to distinguish as they are crafted to hide within the nominal internet traffic for complicating detection.

### Validation with Usage Notes - A

We validate the dataset with a machine learning evaluation that follows the steps in the section Usage Notes – A.

As intrusion detectors, we train and run two different popular machine learning algorithms, namely, the unsupervised Isolation Forests^42^, and the supervised XGBoost^43,44^.

Given True Positives (TP) and True Negatives (TN) as the correctly predicted values, and False Positives (FP) and False Negatives (FN) as the misclassified events, the accuracy measures the correct predictions in a classification task, and it is defined as A = (TP + TN) / (TP + TN + FP + FN). We evaluate the performance using the accuracy, the Receiver Operating Characteristic curve (ROC), and the precision-recall curve. The ROC curve plots the probability that the model will correctly rank a randomly chosen positive instance higher than a random negative one^45,46^: it represents the trade-off between recall and False Positive Rate, where recall is R = TP / (TP + FN), and the false positive rate is FPR = FP / (TP + FP). Further, we use the Precision-Recall curve which calculates the trade-off between precision and recall for different detection thresholds, where precision is P = TP / (TP + FP). In Table 6, we report the performance of the Isolation Forest and XGBoost obtained by applying the methodology described in Usage Note A.

**Table 6:** Performance metrics for Isolation Forest and XGBoost obtained by applying the Usage Notes-A.

model	A	FPR	R	P
Isolation Forests	0.875	0.057	0.895	0.942
XGBoost	0.996	0.0018	0.997	0.998

We report on the accuracy (A), the False Positive Rate (FPR), the Recall (R), and Precision (P).

**Fig. 8:**
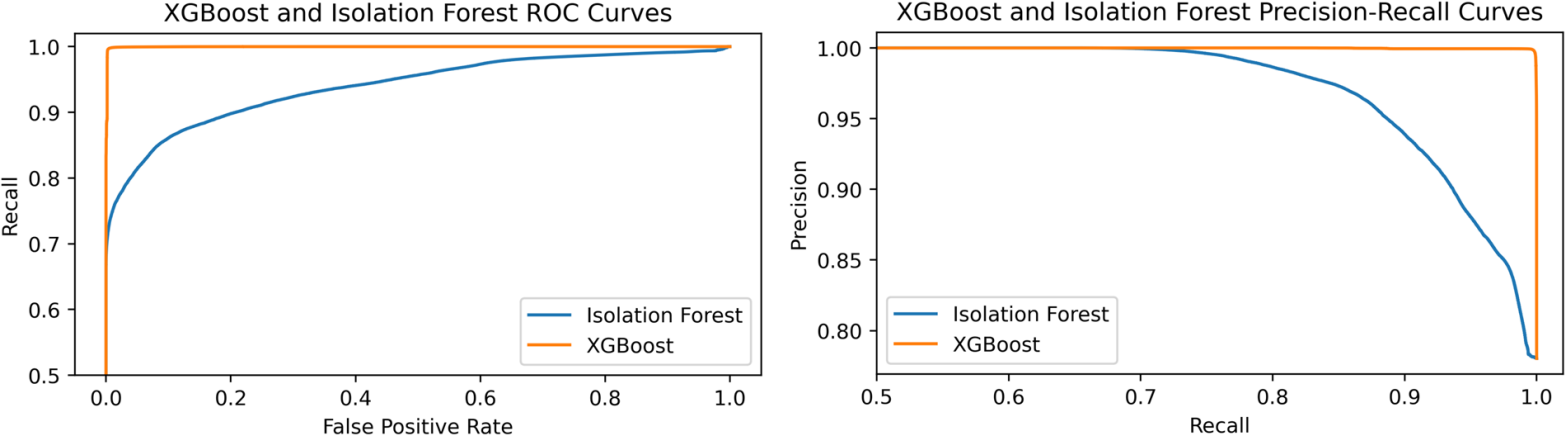
ROC curve and Precision-Recall curve of XGBoost and Isolation Forest on the ROSPaCe^7^ reduced dataset.

In Fig. 8, we detail the ROC curve and the precision-recall curve for Isolation Forest and XGBoost. Their capability to detect attacks validates the usability of our dataset: attacks have a visible effect on the monitored system features, and the dataset can be used to train an anomaly-based intrusion detector. In addition, we observe that the ROC and precision-recall curves show opposite and regular trends, assuring that the accuracy result is not biased by inter-class imbalance.

### Validation with Usage Note – B

In this section, we report the results of the machine learning validation we perform by leveraging the sequence structure of the dataset as detailed in the section Usage Notes - B.

We take advantage of the ROSPaCe^7^ characteristics to evaluate the attack latency, i.e., measure how long an attack will be perpetrated in the system before being detected. The metrics from Usage Note - A may present a misleading understanding of the actual capability to defend from attacks. For example, accuracy seems satisfactory, but the detector may have missed the initial part of the attack, which still might be sufficient to reach determined attack goals.

We resort to the organization in blocks of the test set produced in Usage Note-B. For each block, we keep track of two data points: (i) the first attack data point, and (ii) the first attack data point that is correctly classified. The attack latency is the distance between the two data points: it can be measured as a time interval, or as the number of data points that occurred between the two attack data points.

In addition, we also are interested in understanding the number of false positives, which is identified by the FPR. A high FPR means many false alarms, which ultimately make the system unusable: consequently, we decide on a maximum acceptable FPR, and we compute the recall and the attack latency. In this paper, we set the maximum FPR = 10^−4^, which means that during the normal operativity, one data point every 10^4^ data points is erroneously classified as an attack. We are aware that, in practice, the FPR threshold should be agreed upon with SPaCe engineers and security managers, but the deployment of an IDS is outside the scope of the paper.

As an algorithm, we use XGBoost to classify each data point in a block. Time series algorithms, like long short-term memory (LSTM^34^) recurrent neural networks, could also be exploited; however, a comparison of the most suitable algorithm for ROSPaCe^7^ is outside the scope of the paper.

We report the performances of XGBoost in Table 7, Table 8, and Table 9. Table 7 describes the attack latency in terms of seconds elapsed from the start of the attack until the first detection. Table 8 presents the number of consecutive false negatives (misclassified attack data points) from the start of the attack sequence until the first detected attack. Table 9 reports the number of blocks where there is at least one detected attack.

**Table 7:** Attack latency, in seconds.

attack	average detection latency	standard deviation	minimum detection latency	maximum detection latency
flooding meta	13.08	19.58	0.18	126.80
nmap discovery	6.74	4.14	0	13.16
flooding nmap	0.18	0.09	0.04	0.35
ROS2 node crashing	0.05	0.04	0.01	0.09
ROS2 reconnaissance	5.63	2.58	2.33	8.46
ROS2 reflection	20.10	28.38	0.03	40.17

We provide the minimum value, the maximum value, and the standard deviation.

**Table 8:** Attack latency, in terms of the number of data items that occurred before detection.

attack	average detection index	standard deviation	minimum detection index	maximum detection index
flooding meta	2 111	3 196	14	16 245
nmap discovery	167	350	0	3 613
flooding nmap	906	3	4 229	1 637
ROS2 node crashing	29	11	16	37
ROS2 reconnaissance	943	424	397	1 420
ROS2 reflection	22	6	18	27

We provide the minimum value, the maximum value, and the standard deviation.

**Table 9:** Ability to detect at least one attack in a block.

attack	total blocks	blocks with detections	detection ratio
flooding meta	105	65	0.62
nmap discovery	162	160	0.99
flooding nmap	8	8	1.0
ROS2 node crashing	40	3	0.07
ROS2 reconnaissance	78	5	0.12
ROS2 reflection	78	2	0.02

We report the total number of blocks in the test set, where at least an attack is detected, and the corresponding detection ratio.

Aggregating the results from the different tables, is evident that we can detect Flooding meta, Nmap discovery, and Flooding nmap, while we show a very low capability in detecting the three ROS2 attacks. These attacks are less represented in the dataset having shortest sequences with a smaller number of packages, because they lead the system in a failed state.

We observe that all the Flooding nmap blocks are detected (Table 9), with a very short detection latency: the average latency is 0.18 seconds (Table 7), during which 906 packets are received (Table 8). Somehow good results are obtained with nmap discovery, where we can detect at least one attack data point in 99% of the attack sequences. However, attack latency is significantly higher (6.74 seconds), but it is a direct consequence of the differences between the two attacks. The other attacks are more difficult to detect, and especially the ROS2-specific attacks, which have a very low detection ratio in Table 9. This suggests that the effect of ROS2 attacks on the monitored features is rather different from one block to another, and XGBoost is not able to generalize sufficiently to capture this difference. While this may look like a concern for the development of efficient intrusion detectors, these insights can serve as a foundation for developing a new approach for training and fine-tuning an intrusion detection system.

## Usage Notes

The steps delineated in this section are aimed at arranging the data into a structure that is well-suited for efficient and effective utilization. The steps are implemented in two *Jupyter* notebooks available in our software repository^47^, and they can be executed straightforwardly only at the cost of setting the correct path to the dataset file.

First, we reduce to numeric all the features with mixed data types (e.g., alphanumeric, and numeric values). Second, we replace *nan* values and infinite values with −1 as they are usually not accepted by ML algorithms. Then, we map attack labels to numeric values: to distinguish between normal data points and attack data points, we just need binary classification and consequently, we transform each attack label to 1 and each normal label to 0. Third, we convert string values to numbers using categorical encoding. This technique assigns a unique number to each unique string value of a feature. After implementing this preprocessing, the dataset can be used for two purposes: to measure the ability to distinguish between normal and attack data points and to measure the attack latency. Each approach relies on the application of additional preprocessing steps, described respectively in the Usage Notes-A and Usage Notes-B below.

### Usage notes – A

Our focus is to distinguish between normal and attack data points: therefore, we drop all the time- and sequence-related features, and we shuffle the dataset. Timestamps or time-related data in the dataset can inadvertently serve as labels, potentially leading to misleading results. Very practically, the steps are:

1. Drop all columns containing timestamps or that may contain information on data sequences.
2. Shuffle the dataset.
3. Divide the dataset into train and test sets by applying a 60/40 split.
4. Train and test a binary classifier, compute the appropriate metrics (see the Technical Validation below), and visualize results.

### Usage notes – B

In this scenario, we shift our focus to investigate how long an attacker is undetected. The notion of time, in this case, is central: we want to train the ML detector to make decisions based on system evolution through time rather than deciding on individual data points. Therefore, the time-related features must be included in the dataset to relate subsequent data points. As the dataset is already organized in alternating blocks of normal behavior and attack scenarios, the only steps needed are the following:

1. Identify all the pairs (normal sequence, attack sequence) in the dataset. We call each of these pairs a *block*.
2. 60% of randomly selected blocks are assigned to the train set, and the remaining 40% to the test set. This way we train the ML detector with blocks of ordered data points, each block containing approximately 30 seconds of normal data, that are followed by a variable amount of attack data.
3. Train and test a binary classifier, compute the appropriate metrics (see the Technical Validation below), and visualize results.

## Data Availability

The software code to run the entire experimental campaign can be downloaded from the *codes* folder of our open software repository^47^. Each subfolder corresponds to a specific activity of the dataset creation procedure reported in this paper. The ROSPaCe proposed versions are available in a public repository and can be freely downloaded. The repository contains a folder for each of the datasets, and an additional CSV file that enlists the features included.
